# Laparoscopic management of an unexpected cholecystogastric fistula during elective cholecystectomy: a case report

**DOI:** 10.1093/jscr/rjag402

**Published:** 2026-05-29

**Authors:** Silvana Alexandra Valencia Valverde, Esteban Alexander Caiza Molina, Miguel Ángel Moyón, Mateo Sebastián Valencia Villalba

**Affiliations:** Department of Surgery, General Hospital San Francisco, Quito, 170120, Ecuador; Estudiante de posgrado en Cirugía General, Universidad de las Américas, Quito, Ecuador; Department of Surgery, General Hospital San Francisco, Quito, 170120, Ecuador; Universidad Tecnológica Equinoccial, Quito 170147, Ecuador

**Keywords:** cholecystogastric fistula, laparoscopy, cholecystectomy, minimally invasive surgery, biliary complications

## Abstract

Cholecystogastric fistula (CGF) is a rare complication of chronic cholelithiasis characterized by an abnormal communication between the gallbladder and the stomach. We report the case of a 69-year-old female who underwent elective laparoscopic cholecystectomy for symptomatic gallstone disease. Intraoperatively, a CGF was unexpectedly identified due to dense adhesions between the gallbladder and the gastric antrum. The fistula was successfully managed laparoscopically using a linear endostapler for resection and primary closure of the gastric defect, followed by standard cholecystectomy. The patient had an uneventful recovery and was discharged within 24 hours. This case highlights the feasibility and safety of laparoscopic management of CGF in experienced hands while emphasizing the importance of intraoperative adaptability.

## Introduction

Cholecystogastric fistula (CGF) is a rare condition characterized by an abnormal communication between the gallbladder and the stomach, typically arising as a complication of chronic cholecystitis secondary to gallstone disease. Diagnosis is challenging due to its nonspecific clinical presentation, resulting in low preoperative detection rates [1].

The prevalence of cholelithiasis is ~15%, with up to 20% of patients developing symptoms within 5 years. These cases may progress to more severe conditions such as acute cholecystitis, choledocholithiasis, or biliary pancreatitis [1, 2]. The incidence of bilioenteric fistulas in patients with gallstones ranges from 0.15% to 8% [3].

Bilioenteric fistulas develop as a result of chronic inflammation and pressure-induced erosion of gallstones into adjacent gastrointestinal structures and are considered a late manifestation of Mirizzi syndrome [4]. They are classified as cholecystoduodenal (40%), cholecystocolonic (28%), and cholecystogastric fistulas (32%) [4].

Preoperative diagnosis remains difficult due to the absence of specific clinical signs. Computed tomography findings may suggest the diagnosis by demonstrating close apposition of adjacent organs, inflammatory changes, ectopic gallstones, or direct visualization of the fistulous tract [1]. In most cases, diagnosis is made intraoperatively.

## Case report

A 69-year-old female from Otavalo, Ecuador, presented with a 3-year history of intermittent right upper quadrant pain consistent with biliary colic. There was no history of jaundice, gastrointestinal bleeding, or weight loss. Laboratory tests were within normal limits, and abdominal ultrasound revealed cholelithiasis without signs of acute inflammation.

**Figure 1:**
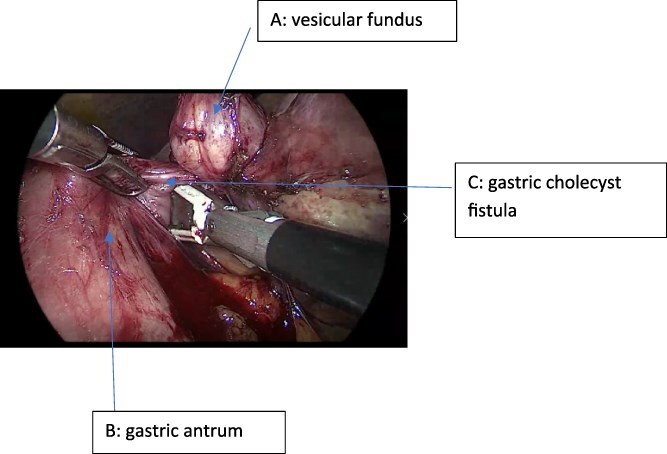
Intraoperative view showing the gallbladder fundus, the cholecystogastric fistula (CGF), and the gastric antrum.

The patient was scheduled for elective laparoscopic cholecystectomy. During surgery, dense adhesions were identified between the gallbladder and the gastric antrum (Fig. 1). Careful dissection revealed a cholecystogastric fistula, an uncommon intraoperative finding requiring advanced laparoscopic expertise [5, 6].

A laparoscopic approach was performed. Trocars were placed as follows: two 5 mm trocars in the right flank and right hypochondrium, one 11 mm subxiphoid trocar, one 11 mm umbilical trocar, and one 12 mm trocar in the left hypochondrium to allow the insertion of a 60 mm linear endoscopic stapler.

**Figure 2:**
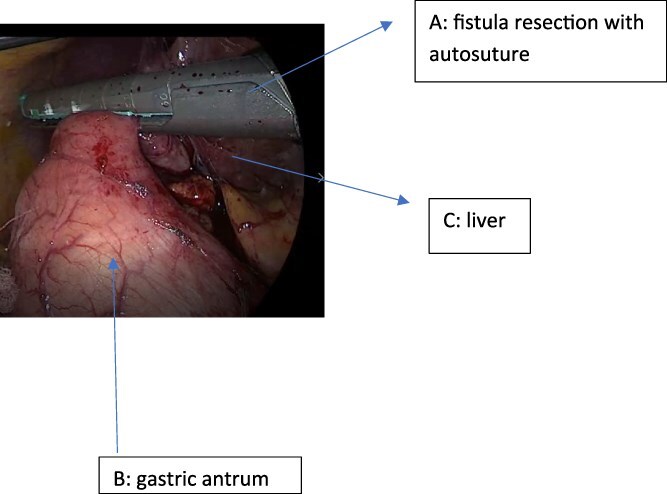
Resection of the cholecystogastric fistula using a 60 mm green-load linear endostapler.

The fistulous tract was meticulously dissected and completely isolated using an energy device (LigaSure). The fistula was then divided using a linear endoscopic stapler with a green cartridge, achieving simultaneous resection of the tract and primary closure of the gastric defect (Fig. 2).

Subsequently, a standard laparoscopic cholecystectomy was performed. The gallbladder fundus was retracted cephalad through the right flank port, while Hartmann’s pouch was retracted laterally to expose Calot’s triangle. Careful dissection allowed clear identification of the cystic duct and cystic artery. Both structures were clipped with two proximal and one distal Hem-o-lok clips and then divided.

**Figure 3:**
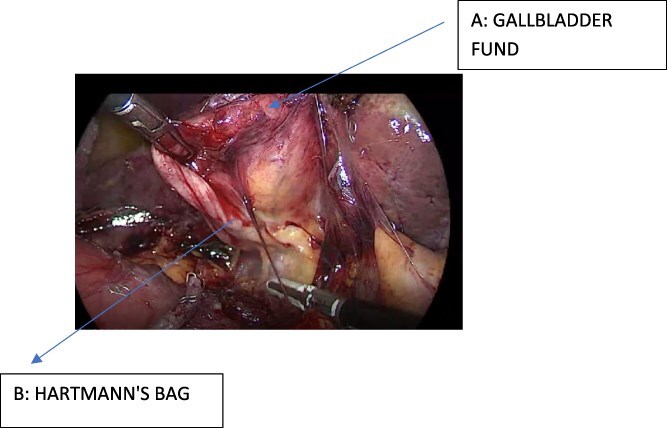
Intraoperative view after fistula resection, showing the gallbladder fundus and Hartmann's pouch.

A retrograde (fundus-first) cholecystectomy was completed. Hemostasis was confirmed, and a closed-suction drain (Jackson-Pratt) was placed in the surgical bed (Fig. 3).

The postoperative course was uneventful. The patient demonstrated adequate pain control, tolerated oral intake early, and was discharged 24 hours after surgery. At follow-up, she remained asymptomatic.

## Discussion

CGF is a rare entity often diagnosed intraoperatively due to its nonspecific presentation [1]. Chronic inflammation and gallstone erosion are the main mechanisms involved in fistula formation [4].

Preoperative diagnosis remains difficult, and imaging studies frequently fail to detect the condition [1, 2]. Therefore, surgeons must be prepared to manage unexpected intraoperative findings.

Traditionally, these fistulas were managed through open surgery due to the complexity of the anatomy and the presence of dense adhesions. However, advances in minimally invasive techniques have made laparoscopic management a feasible and safe alternative in selected patients [3, 7, 8].

In the present case, the use of a linear endoscopic stapler enabled safe division of the fistula and effective closure of the gastric defect. This technique offers advantages over hand-sewn repair, including shorter operative time and a potentially lower risk of leakage when appropriately applied. In similar cases, some surgeons may opt for conversion to open surgery; however, with adequate technique and meticulous dissection, advanced procedures can be safely accomplished laparoscopically in experienced hands.

The laparoscopic approach also provides well-established benefits, including reduced postoperative pain, shorter hospital stay, and faster recovery. However, careful patient selection and intraoperative judgment remain critical.
